# Management Strategies in Cardiac Surgery for Postoperative Atrial Fibrillation: Contemporary Prophylaxis and Futuristic Anticoagulant Possibilities

**DOI:** 10.1155/2013/637482

**Published:** 2013-12-05

**Authors:** George Tokmaji, R. Scott McClure, Tsuyoshi Kaneko, Sary F. Aranki

**Affiliations:** Division of Cardiac Surgery, Brigham and Women's Hospital, Harvard Medical School, 75 Francis Street, Boston, MA 02115, USA

## Abstract

With more than a third of patients expected to endure the arrhythmia at any given time point, atrial fibrillation after cardiac surgery becomes a vexing problem in the postoperative care of cardiac surgery patients. The impact on patient care covers a spectrum from the more common clinically insignificant sequelae to debilitating embolic events. Despite this, postoperative atrial fibrillation generally masquerades as being insignificant, or at most as an anticipated inherent risk, merely extending one's hospital stay by a few days. As an independent risk factor for stroke, early and late mortality, and being a multibillion dollar strain on the healthcare system annually, postoperative atrial fibrillation is far more flagrant than a mere inherent risk. It is a serious medical quandary, which is not recognized as such. Though complete prevention is unrealistic, a step-wise treatment strategy that incorporates multiple preventative modalities can significantly reduce the impact of postoperative atrial fibrillation on patient care. The aims of this review are to present a brief overview of the arrhythmia's etiology, risk factors, and preventative strategies to reduce associated morbidities. Newer anticoagulants and the potential role of these drugs on future treatment paradigms are also discussed.

## 1. Introduction

Atrial fibrillation (AF) is the most common arrhythmia and morbidity after cardiac surgery. Though the incidence varies depending on the intensity of monitoring, best estimates suggest that nearly 30% of patients undergoing coronary artery bypass grafting (CABG) surgery, 40% of patients undergoing valvular heart surgery, and more than the half of all patients undergoing combined coronary and valvular procedures will develop the arrhythmia [1, 2]. Although postoperative atrial fibrillation (POAF) is at times dismissed as a nonissue due to its often benign course, POAF remains a serious medical concern. The arrhythmia poses serious risks to patients in the postoperative period and requires countless preventative healthcare expenditure [3–10]. This paper is an up-to-date look into POAF etiology, risk factors, and consequences. Treatment strategies to reduce the incidence of POAF and preventative modalities to minimize risk of the arrhythmia are also discussed. There is currently no single treatment or preventative option for POAF. A systematic approach that is initiated in the preoperative period and continued to the perioperative recovery phase offers the best preventative strategy. Futuristic anticoagulants and their potential impact on hospital length of stay and associated hospital costs are also discussed.

## 2. Etiology

Though our understanding of the biochemical and cellular interplays of POAF remains incomplete, the multiple wavelet re-entry theory has proven a useful model and is generally regarded as the predominant process [11–13]. Other models, such as the focal mechanism theory and the mother rotor theory, have also been described [12–14].

The multiple-wavelet theory hypothesizes that AF is sustained by multiple, equally dominant, concurrent, and re-entry circuits, due to an alteration or change in the atrial substrate. This change in the substrate subsequently slows the propagation of the forward moving action potential through the atrial tissues and results in a unidirectional block. This phenomenon occurs in conjunction with a shortening of the refractory period in alternate directions causing the impulse to take a retrograde course. This single event occurs in countless repetition creating multiple re-entrant wavelets through the surmised “atrial dispersion of refractoriness”. These re-entrant wavelets produce an electrically unstable environment within the atria that are highly susceptible to AF. Once present, a triggering event, some initiating force (i.e., premature atrial contraction) sets the process of AF in motion. Both the initiating trigger and an altered substrate to sustain the arrhythmia are required for AF to occur. Specific to cardiac surgery, there are several variables throughout the surgical period where both, a triggering event and an alteration to the atrial substrate, could occur meeting the necessary conditions for AF to formulate.

The focal mechanism theory hypothesizes that AF is sustained by rapidly firing focal discharges from the atria, most likely generated in sleeves of the pulmonary vein [15]. Several mechanisms of ectopic impulse generation (focal discharges) have been described. These include enhanced automaticity, delayed afterdepolarizations (DADs), and early after repolarizations (EADs). These rapid ectopic beats are dependent upon the slope of phase 4 depolarization. Phase 4 depolarization is the period required to attain threshold potential thus creating an action potential. The slope of phase 4 could become elevated due to an increased atrial expression of ion channel subunits. When this occurs, the spontaneous rate enhances, thus increasing the risk of ectopic discharges or sustained AF.

Moreover, a distortion in the cellular calcium homeostasis, especially calcium overload, can develop DADs. Delayed afterdepolarizations are impulsive hump-shaped depolarizations that occur after complete repolarization. Of note, the mechanism and form of DADs are different from spontaneous phase 4 depolarization. If these DADs reach the threshold potential, cell firing occurs either as a single ectopic beat or as a sustained atrial tachycardia. Calcium enters the cells through calcium channels with each separate action potential. Rapid firing rates increase calcium entry and therefore potentially provoke DAD-related arrhythmias.

In addition, EADs that occur during repolarization may also induce triggered activity. Early afterdepolarizations result from action potentials that become unusually extended, which allow calcium channels (*I*_CaL_) to recover from inactivation and generate abnormal depolarizations at plateau potentials [13].

One of the more novel hypotheses regarding the mechanism of AF is the mother rotor theory. Rotors are described as key drivers or organizing centres of fibrillation [14]. Unlike the multiple-wavelet theory, which suggests that AF results from multiple, equally dominant, and re-entrant circuits, the mother rotors theory claims that one chief core “mother re-entrant wave” discards and instigates several “daughter waves”. Atrial fibrillation is sustained by the mother rotor, where daughter wavelets originate but disseminate in an irregular pattern due to fibrillatory conduction. This fibrillatory conduction is caused by a wave break in anatomically determined areas in the atria including pectinate muscles [14]. It could therefore be plausible that a rotor or even several rotors are the main drivers that sustain POAF.

Still, from a clinical perspective in the postcardiac surgery patient, regardless of the exact pathway involved, alteration in the atrial tissue from the combined impact of a patient's preoperative characteristics and perioperative insults is inevitably thought to be responsible for POAF.

**Figure 1:**
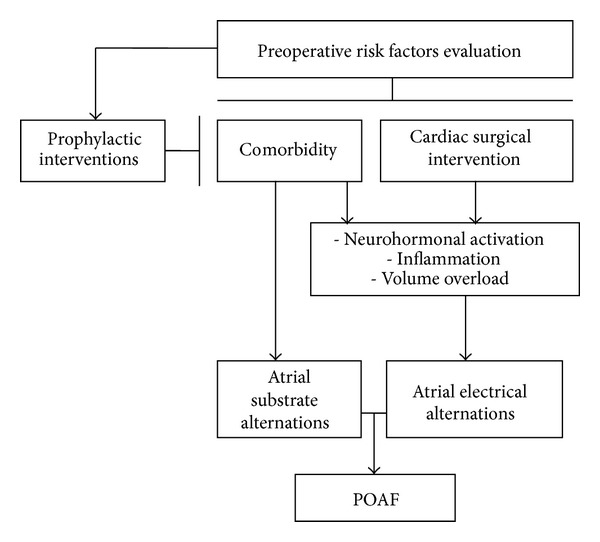
Schematic illustration of several factors that contribute to POAF. (postoperative atrial fibrillation = POAF).

Certain biochemical mechanisms either trigger POAF or affect the atrial substrate and sustain POAF. Neurohormonal activation, volume overload, and the complex biochemical by-products of inflammation each has a role in this regard.  Figure 1 illustrates a schematic overview of several factors that contribute to the pathogenesis of POAF.

Neurohormonal activation and an increase in sympathetic stimulant often precede AF implicating this biochemical pathway as a trigger. Clinically, pain, intraoperative heart manipulation, hypovolemia, or any additional perioperative insult causing an upregulation in catecholamines can then activate adrenoreceptors within the left atrium and heighten their propensity to engage an electrical response or trigger an event [16].

Conversely, persistent volume overload is thought to sustain POAF. Increased volume alters atrial pressures and atrial compliance. Volume overload has proven to shorten atrial refractory periods in the atria of animals and humans, similarly, lending support to its role in changing the atrial substrate [17, 18].

Clinical evidence supports the relationship between increased volume and POAF. In a prospective series of patients undergoing CABG, preoperative left atrial volume as determined by echocardiography was shown to be an independent risk factor for the occurrence of POAF [19]. Moreover, in the second atrial fibrillation suppression trial, a net fluid balance on postoperative day 2 was an independent predictor of POAF [20].

Evidence to implicate inflammation as a major pathogenesis of POAF continues to build. A recent study of 104 patients undergoing on-pump CABG noted a significant increase in intraoperative inflammatory plasma biomarkers prior to cardiopulmonary bypass (high-sensitivity C-reactive protein and interleukin-6) acquired from the atria of patients who had POAF as opposed to the patients that remained in normal sinus rhythm [21]. The study suggests that a pre-existing intracardiac inflammatory environment predisposes a patient to POAF. In a canine model, inflammation has proven to slow the electrical conduction velocity of the action potential traversing atrial tissue, which supports the re-entrant wavelet model [22]. Slowing the conduction velocity results in unidirectional block and sets the stage for re-entrant wavelets as previously discussed.

These are only a few examples amidst a conglomerate of conjoined variables in what is known as a complex multifactorial process inevitably responsible for causing POAF.

## 3. Risk Factors

While the underlying biochemical and cellular mechanisms involved in POAF are highly complex and remain incompletely understood, at the clinical level there are easily identifiable risk factors that predispose patients to POAF. These risk factors can be divided into three phases, namely, preoperative, intraoperative, and postoperative management. Moreover, these risk factors can be categorized as either being modifiable or nonmodifiable (Table 1) [3, 10, 23–27]. This simple schema aids in formulating preventative management strategies.

Although there are inconsistencies with respect to some risk factors in the published literature, advanced age is the single most important and consistently proven factor to increase a patient's risk of POAF [23, 28]. Age has shown to increase the risk of POAF 1.5-fold for each 5-year increment starting at age 50 up to age 75 [29]. Similarly, in a large multicenter observational study of 4600 patients, age was noted to increase the risk of POAF by 75% for each 10-year increment starting at age 40. The very same study concluded that all patients aged 70 or older are at high risk for POAF [3].

The various intraoperative and postoperative risk factors responsible for POAF impose their effects through one of the following mechanisms: either an upregulation of the body's sympathetic response, a slowing of the conduction velocity with atrial stretch or by inducing an inflammatory cascade. Myocardial ischemia, atrial cannulation, aortic cross-clamp time, left ventricular venting, hyper and hypovolemic states, damage to the atrium, endotracheal tube insertion, excess inotropic requirements, and electrolyte imbalances are snapshots into the various factors published in the literature as potential predictors for POAF [3, 10, 11, 16, 17, 30]. Extracorporeal circulation and its impact on POAF in CABG are important topics that remain controversial. Several meta-analyses have consistently shown substantial reductions in the occurrence of POAF with off-pump CABG in comparison to on-pump procedures [24, 31, 32]. However the largest randomized controlled trial (RCT) to date comparing outcomes between on and off-pump CABG procedure on over 4,700 patients showed no difference in POAF amongst the two groups (OR 1.02; 95% CI 0.9 to 1.15; *P* = 0.72) [33].

## 4. Morbidity, Mortality, and Anticoagulant Therapy

Generally, POAF is considered a transient, self-limiting event that is well tolerated with no discernible effects during the postoperative course. However, this is not always the case, as the presence of POAF can have a deleterious effect on patient recovery and impact patient care in a variety of ways. Patients may experience fatigue, palpitations, or diaphoresis, which is of significant discomfort for them. Or at the other extreme, acute hypotension and hemodynamic instability may be the initial presentation, necessitating immediate cardioversion [34]. Most importantly, POAF increases one's thromboembolic load which is associated with stroke risk and increases a patient's mortality.

Strokes can be catastrophic and significantly impact quality of life and long-term survival [35]. The incidence of stroke in patients who develop POAF following cardiac surgery is 3-fold higher than it is in patients who remain in normal sinus rhythm [25]. Moreover, not only does an early postoperative stroke affect early mortality, but it also affects late mortality as well [30]. In a study of more than 16,000 patients undergoing CABG, POAF was also found to incur a 21% relative increase in late mortality (mean 6-year followup) [10]. Similar results have been reported in a recent observational study from Australia on over 19,000 patients [36]. This would suggest that the presence of the arrhythmia is at the very least a marker for long-term mortality.

The cornerstone of treating POAF is the preventative strategy that will increase the likelihood of maintaining normal sinus rhythm for the duration of the postoperative course. These varied preventative strategies are discussed later in the paper.

For patients who fail preventative therapy, standard treatment with rate control and anticoagulation should be instituted to minimize complications. In general, the treatment of patients with POAF is similar to treatment of nonsurgical patients who develop AF. The primary goal is anticoagulation with rate control. The atrial fibrillation follow-up investigation of rhythm management trial (AFFIRM), a clinical trial on over 4,000 patients, showed that there was no difference in mortality between patients with chronic AF treated with anticoagulation and rate control in comparison to anticoagulation and rhythm control (HR 1.15; 95% CI 0.99 to 1.34; *P* = 0.08). The utilization of antiarrhythmic agents for rhythm control was associated with more drug induced adverse effects including an increased risk for cardiac depression and torsade de pointes [37]. The American College of Cardiology Foundation/American Heart Association Task Force on Practice Guidelines (ACC/AHA) therefore recommends the administration of AV nodal blocking agents to achieve rate control in patients who develop POAF (class I; level B) [34].

When POAF persists for more than 48 hours, both the American College of Chest Physicians and the American College of Cardiology Foundation recommend anticoagulation for 30 days with a vitamin K antagonist (warfarin) to achieve an international normalizing ratio between 2.0 and 3.0 [34, 38]. Bridging with intravenous heparin is only recommended for high-risk patients who had a prior thromboembolic event. These recommendations need to be implemented in the context of each individuals bleeding risk with respect to anticoagulation administration in the postoperative period [34, 38].

Warfarin therapy is the long-term anticoagulant of choice for POAF and has remained so since its introduction. Although it has proven to be effective at reducing the risk of stroke, warfarin's variable dose response, narrow therapeutic index, various drug and food interactions, frequent testing requirements, and poor patient compliance are some of the medications many shortcomings. New oral anticoagulants with more favourable risk profiles, specifically the direct thrombin inhibitor, dabigatran, and the direct factor Xa inhibitor, rivaroxaban and apixaban, were recently approved by Food and Drug Administration (FDA) in the United States for the treatment of stroke and systemic embolism in nonvalvular atrial fibrillation. In a clinical trial where 18,000 patients with nonvalvular AF were randomized to dabigatran or adjusted-dose warfarin, twice daily dosing of dabigatran at 150 mg reduced the risk of stroke or peripheral embolic events by 34% (RR 0.66; 95% CI 0.53 to 0.82; *P* < 0.001) while showing no increase in bleeding risk (RR 0.93; 95% CI 0.81 to 1.07; *P* = 0.31) [39]. In addition to this, dabigatran does not demonstrate an interaction with other drugs, does not necessitate regular monitoring, and has a short half-life. Based largely on this data, dabigatran has recently been given a class I recommendation by the American College of Cardiology Foundation for the treatment on nonvalvular AF [40]. Similarly, rivaroxaban at a once daily dose of 20 mg reduced the risk of stroke and peripheral embolic events by 21% (RR 0.79; 95% CI 0.66 to 0.96) with similar bleeding profiles when compared to adjusted-dose warfarin in a RCT of 14,000 patients with nonvalvular AF [41]. Also, a RCT (ARISTOTLE trial) with a mean follow-up of 1.8 years involving 18,201 patients with AF and at least one additional risk factor for stroke were randomized to receive either apixaban at a twice daily dose of 5 mg or warfarin (INR 2.0 to 3.0). Up to 62% of the patients achieved the therapeutic INR range. In addition, both cohort arms permitted up to 162 mg of aspirin. The trial demonstrated a significant reduction in favour of apixaban with regard to stroke or systematic embolism (HR 0.79; 95% CI 0.66 to 0.95 *P* = 0.01), major bleeding (HR 0.69; 95% CI, 0.60 to 0.80; *P* < 0.001), and mortality (HR 0.89; 95% CI, 0.80 to 0.99; *P* = 0.047) [42].

However, none of these trials included patients undergoing cardiac surgery. To that end, neither the results from these trials can be generalized to the management of POAF in cardiac surgery nor have these anticoagulants been approved for such an indication. Nonetheless, the results from these trials pave the way for future studies to assess the risk-benefit ratio of these medications for the cardiac surgery population. Since the time required to achieve therapeutic levels of warfarin is known to significantly increase hospital length of stay, one could speculate that the use of these newer anticoagulants with their immediate onset of action could substantially reduce hospital stay and in turn reduce hospital costs [23]. Moreover, if these new anticoagulants could prove to reduce stroke and bleeding in the POAF population, the reduction in costs could be substantial. Yet, the current cost of dabigatran is 18 times that of warfarin ($9/day versus $0.50/day), which would mitigate some of its cost-effectiveness.

## 5. Preventative Strategies

Pharmacotherapy is the mainstay preventative measure to reduce the incidence of POAF. Several different drugs have been utilized in various combinations and studied at various stages in the perioperative period with different doses and routes of administration. The various combinations have made it difficult to find the best approach for preventing POAF. Currently, *β*-Blockers, amiodarone, and sotalol are the three most commonly used medications to prevent POAF.

Upstream therapies including statins, magnesium, and corticosteroids are less widely used but there is evidence to support their utilization. Several intraoperative prophylaxes have also been utilized to reduce the incidence of POAF, including atrial pacing and posterior pericardiotomy. Tables 2, 3, and 4 select the best evidence meta-analyses and RCTs to support the use of these medications and nonpharmaceutical interventions.

### 5.1. Nonpharmacological Prophylactic Interventions

#### 5.1.1. Posterior Pericardiotomy

Non-pharmacologic interventions have become attractive preventative strategies for AF after cardiac surgery because of the absence of drug-induced adverse events. Posterior pericardiotomy is an intraoperative intervention in which generally a 4 cm longitudinal incision is made posterior in the pericardium and parallel to the phrenic nerve. Blood effusion in the pericardium could induce cardiac irritation which is a risk factor for developing POAF. The rationale for this procedure is mainly that this technique allows postoperative fluid drainage, thus preventing pericardial effusion [26, 27]. A meta-analysis of 6 studies involving 763 patients showed a significant reduction in POAF with an absolute risk reduction of 19% in the posterior pericardiotomy group (OR 0.35; 95% CI 0.18 to 0.67; *P* = 0.001; Number needed to harm (NNH) = 6) [43]. Another meta-analysis of 6 studies in 763 patients undergoing a CABG surgery confirmed this result (OR 0.33; 95% CI 0.16 to 0.69; *P* = 0.003; NNT = 6) [44]. However, posterior pericardiotomy was not associated with reduction in postoperative mortality rate or length of hospital stay [43].

A potential complication of this surgical procedure is the possible risk of cardiac herniation. Bypass grafts after a CABG procedure can obtrude and could potentially be compressed by the edges of the posterior pericardiotomy. A partial posterior pericardiotomy incision distant from the bypass graft limits the risk of this complication. [59]. There is also a risk of phrenic nerve injury which can be limited by direct visualization.

The present data shows that posterior pericardiotomy is an effective method, although the evidence level is not strong. It also lacks other morbidity data such as stroke, reoperation, postoperative hemodynamic instability, and pericardial effusion. Therefore, it still remains controversial whether this surgical procedure should be utilized as a standard prophylactic intervention.

#### 5.1.2. Atrial Pacing

Atrial pacing controls the cardiac rate via electrical stimulation. Prevention of POAF could be achieved by restraining atrial premature beats. Unlike pharmacological prophylaxis, pacing prophylaxis is not associated with the risk of developing bradycardia or hypotension. Additionally, pacing therapy has the advantage that it does not need to be initiated before cardiac surgery in contrast to most of the pharmacological prophylaxis.

Multiple pacing strategies have been utilized to prevent POAF. These include left atrial pacing, right atrial pacing, biatrial pacing, and Bachmann's bundle pacing. A meta-analysis of 14 studies in 1846 patients analyzed the relationship between atrial pacing and prevention of POAF. Only biatrial pacing showed a significant result with regard to shorter length of stay and risk reduction of POAF (OR 0.44, 95% CI 0.31 to 0.64; *P* < 0.001; NNT = 7). Other pacing configurations did not show a significant outcome in reducing POAF [45].

A more recent meta-analysis of 21 studies involving 2633 patients showed a favourable outcome for atrial pacing with regard to prevention of POAF and length of stay (OR 0.47; 95% CI 0.36 to 0.61; *P* < 0.00001; NNT = 8). This meta-analysis was a pooled analysis of all atrial pacing techniques. Despite these results, atrial pacing was not associated with risk reduction for stroke, cardiovascular mortality or a decrease in hospital costs [43, 45]. A potential side effect of pacing therapy is the risk of malfunctioning atrial leads or inappropriate sensing, which could potentially cause proarrhythmic atrial extrastimulation, thus increasing the likelihood of arrhythmias.

Atrial pacing therapy, especially biatrial pacing seems to be an effective strategy in preventing POAF and should therefore be considered an option for prevention of POAF if patients have a contraindication for pharmacological prophylaxis, especially *β*-Blockers and amiodarone therapy.

### 5.2. Pharmacological Prophylactic Interventions

#### 5.2.1. *β*-Blocker Therapy

*β*-Blockers are class II antiarrhythmic drugs and have antiarrhythmic action on both myocardial and stimulus conduction cells. *β*-Blocker therapy is considered the most successful prophylaxis therapy in regard to POAF prevention. A risk factor for developing POAF is the withdrawal of *β*-blocker therapy; therefore this should be avoided whenever possible. A recent meta-analysis of 10 studies involving 2,556 patients who underwent CABG demonstrated a reduction in POAF in the *β*-blocker therapy group (OR 0.50; 95% CI 0.36 to 0.69; *P* < 0.001; NNT = 8). The vast majority of the patients received *β*-Blocker therapy within 24 hours after cardiac surgery [46]. A similar meta-analysis of 33 studies involving 4,698 patients who underwent either valve and/or CABG surgery has also demonstrated that *β*-blocker therapy reduced the incidence of POAF with an absolute risk ratio of 15.4% (OR 0.33; 95% CI 0.26 to 0.43; *P* < 0.00001; NNT = 7) [43]. However, no risk reduction in stroke, postoperative mortality, cardiovascular mortality, and length of hospital stay was observed [43, 45]. The prophylactic effect of *β*-blocker therapy is the highest when given both before and after cardiac surgery when compared to only before or after cardiac surgery [3, 43, 45]. Potential adverse events of *β* blocker therapy include sinus bradycardia (3%), hypotension (1%), decompensate congestive heart failure exacerbation (<1%), and bronchospasm (rare) [43]. Patients with inotropic maintenance, hemodynamic compromise, second or third degree atrioventricular block, or chronic lung disease are generally contraindicated with regard to *β* blocker therapy.

The ACC/AHA Guidelines recommend that in the absence of contraindications, *β*-blocker therapy should be applied to patients undergoing cardiac surgery (class I, level A) [34]. Oral *β*-Blocker therapy should be administrated at least 1 week before cardiac surgery with a *β* ^1^-blocker nonintrinsic sympathomimetic effect if possible (class I, level B) [43, 45].

#### 5.2.2. Amiodarone

Amiodarone is a multichannel (K+, Na+, and Ca2+) blocking agent and is considered a class III anti-arrhythmic drug.

Although the variations in dose, duration, and routes of delivery exist, large meta-analyses demonstrated a beneficial effect of amiodarone therapy in regard to POAF prevention.

A recent meta-analysis of 33 studies on 5402 patients demonstrated a significant reduction with respect to the incidence of POAF with an absolute risk ratio of 14% (OR 0.43 95% CI 0.34 to 0.54; *P* < 0.00001; NNT = 8). Additionally, it demonstrated a shorter length hospital stay in the amiodarone group. Despite these results, no significant risk reduction was observed in stroke, postoperative mortality, and cardiovascular mortality [43].

A recent meta-analysis indicated that the effect of amiodarone in prevention of POAF was independent in regard to the route and timing of drug administration and duration of treatment, when at least 300 mg of intravenous amiodarone was given followed by an administration of a total dose of 1 g [47].

On the other hand, several meta-analyses did report an increased risk of postoperative adverse events including postoperative bradycardia and hypotension (16%). Other known adverse events include increase in serum creatinine (93%), phlebitis of administration site, ventricular arrhythmias (2% to 5%), and hepatotoxicity (3% to 20%) [43]. Adverse effects of amiodarone seem to be related to dose and should therefore be given in the lowest dose for a short period if possible. Regimens containing i.v. amiodarone, initiating prophylaxis during the postoperative period, and regimens with average daily doses exceeding 1 g were all associated with an increased risk of adverse events [60]. In addition, combination therapy of amiodarone and *β* blocker therapy increased the risk of developing these adverse events [60]. The ACC/AHA Guidelines recommend that patients who are at high risk for POAF and who have a contraindication for *β*-blocker therapy should consider having prophylactic amiodarone therapy during the preoperative administration to prevent POAF (IIa, level A).

#### 5.2.3. Sotalol

Sotalol is a nonselective *β*-blocker drug also having a class III action of prolonging repolarization by blocking cardiac K+ channels and decreasing neurohormonal activation.

It has been reported in a recent meta-analysis of 11 studies on 1609 patients that sotalol therapy reduced POAF with an absolute risk reduction of 21.9% compared with placebo (OR 0.34; 95% CI 0.26 to 0.43; *P* < 0.00001; NNT = 5). Up to 55% of the included studies administrated sotalol postoperatively. However, no risk reduction was observed in stroke, cardiovascular mortality, and length of stay [43].

Also, a meta-analysis demonstrated that sotalol therapy is more effective in POAF prevention compared to placebo (OR 0.55; 95% CI 0.45 to 0.67; *P* < 0.001; NNT = 6), no treatment (OR 0.33 95% CI 0.24 to 0.46; *P* < 0.001; NNT = 4), or *β*-Blocker therapy (OR 0.60; 95% CI 0.50 to 0.84; *P* < 0.001; NNT = 12). Preoperative sotalol administration was accompanied with more discontinuation due to drug induced adverse effects and also provided no advantage compared to postoperative sotalol administration [48].

Both meta-analyses reported a significantly shorter length of hospital stay in the sotalol group compared to the control groups [43, 48].

Several known adverse side effects have been reported in patients with sotalol therapy after cardiac surgery, including hypotension (6%), dyspnea (21%), bradycardia (16%), and supraventricular arrhythmia (4%) [43]. Due to the most significant complication, Torsade de pointes, prophylactic sotalol drug therapy for POAF must be used with caution, especially in patients who have electrolyte disturbances, prolonged Q-T interval, and supraventricular arrhythmia [61, 62]. The ACC/AHA Guidelines recommend the utilization of sotalol therapy only in high risk patients and in the absence of contraindications (level IIb, B).

### 5.3. Upstream Therapies

Upstream therapy is defined as the utilization of a nonantiarrhythmic treatment that could alter the atrial substrate or any additional target-specific mechanisms of POAF including inflammation reduction. This is desirable for patients with bradycardia and heart failure who are contraindicated to *β*-blocker therapy. Despite having their own side effects, some of these upstream drug therapies have shown to be effective in reducing the incidence of POAF. Statins (HMG-CO-A inhibitors), corticosteroids, magnesium, and *ω*-3 fatty acids will be discussed (Table 4).

#### 5.3.1. Statins

Statins are lipid-lowering drugs that have a large variety of actions including anti-inflammation abilities. This anti-inflammatory effect may reduce the incidence of POAF. A recent meta-analysis of 11 studies that compared preoperative statin therapy with a control group (no statin medication or placebo) in 984 patients who underwent predominantly CABG surgery showed a significant risk reduction of POAF in the statin group with an absolute risk reduction of 17% (OR 0.40; 95% CI: 0.29 to 0.55; *P* < 0.01; NNT = 7). There were no major or minor perioperative side-effects reported with respect to safety endpoint. However, it failed to demonstrate a significant risk reduction in stroke and postoperative mortality [50]. Other meta-analyses have demonstrated a significant reduction of ICU stay and length of hospital stay in patient who received preoperative statin therapy [50, 52].

Another recent meta-analysis of 9 studies on 933 patients undergoing CABG procedures also showed also a beneficial effect of statin with regard to POAF prevention (OR 0.56; 95%CI 0.45 to 0.69; *P* < 0.0001; NNT = 7) [51]. In addition, this was confirmed in an additional meta-analysis of 8 studies on 774 patients evaluating statin therapy for developing AF after cardiac surgery (OR 0.57; 95% CI 0.45 to 0.72; *P* < 0.0006; NNT = 6). In this same study, a meta-regression analysis showed an association between duration of preoperative statin prophylaxis and POAF reduction with 3% reduction per day (*P* = 0.008). No association was observed between statin dose and risk reduction in POAF (*P* = 0.47) [52].

Currently, the ACC/AHA Guidelines recommend that all patients undergoing CABG procedure should receive statin therapy regardless of their lipid profile in the preoperative period including a direct continuation of statin therapy in the postoperative phase (class I, level A) [63]. Although current guidelines do not particularly recommend the utilization of statin therapy to prevent AF after cardiac surgery, current evidence suggests that preoperative statin therapy is associated with a reduction in risk of developing POAF. Therefore, prophylactic statin administration in all patients who tolerate statins at least a week before cardiac surgery for POAF prevention is validated.

#### 5.3.2. Magnesium

Patients with hypomagnesemia are more at risk of developing AF after cardiac surgery [64, 65]. The utilization of prophylactic magnesium during cardiac surgery could therefore correct the magnesium deficiency and potentially prevent the development of POAF.

A recent meta-analysis of 21 studies involving 2988 patients with the majority receiving CABG demonstrated that magnesium therapy decreased the risk of POAF with an absolute risk reduction of 10% (OR 0.55; 95% CI 0.41 to 0.73; *P* < 0.0001; NNT = 11). While dosing regimens varied amongst the included studies, all studies administrated magnesium therapy intravenously and with the majority of the studies (57%) utilizing magnesium therapy in the intraoperative phase [43]. Identical results were reported in another meta-analysis of 15 studies on patients who underwent a CABG procedure (OR 0.61; 95% CI 0.41 to 0.90; *P* < 0.0001; NNT = 13) [49]. Magnesium therapy did not demonstrate a significant risk reduction in stroke, postoperative mortality, and length of hospital stay [43]. Generally, i.v. magnesium administration is associated with few adverse events when a normal renal function is present [66]. Although current guidelines have not yet recommended magnesium therapy as a standard prophylactic option for the prevention of AF after cardiac surgery, it seems that the utilization of magnesium therapy in the perioperative setting could be considered an option for POAF prevention.

#### 5.3.3. Corticosteroids

Because of the anti-inflammatory properties of corticosteroids, several studies have investigated whether corticosteroids could potentially reduce the risk of AF after cardiac surgery.

A meta-analysis of 18 studies on 1509 patients demonstrated a significant reduction in developing AF after cardiac surgery (RR 0.74; 95% CI 0.63 to 0.86; *P* = 0.0001; NNT = 10). No significant difference was observed between different doses of corticosteroids administration. However, an increased risk was observed in the corticosteroids group compared to placebo with respect to hyperglycemia requiring insulin infusion when a high dose of corticosteroids was utilized (>10,000 mg hydrocortisone; NNT = 9) although no increased risk was observed in postoperative mortality (*P* = 0.16) and infection (*P* = 0.73) [53].

A recent meta-analysis of 17 studies involving 1389 patients who underwent CABG surgery showed a reduction of POAF with an absolute risk of 11% in the corticosteroid group compared to placebo (OR 0.60; 95% CI 0.46 to 0.78; *P* = 0.00016; NNT = 13). Both low and high dose of corticosteroids initiation demonstrated the same effect. A significant reduction was also demonstrated in ICU stay. The utilization of corticosteroids had no significant impact on postoperative mortality, stroke, and infections. However, the authors concluded that because *β*-blocker therapy is associated with a smaller amount of side effects than corticosteroid administration, the distinctive reduction of POAF was not an indication for the utilization of corticosteroid therapy [54].

Conversely, a recent RCT (DECS trial) consisting of 4482 patients who received either intraoperative high-dose dexamethasone or placebo demonstrated no beneficial effect of dexamethasone compared to placebo with respect to risk reduction of POAF (OR 0.94; 95% CI 0.87 to 1.02; *P* = 0.14). However, over half of the included patients in both cohorts were utilizing *β*-blocker and statin therapy before cardiac surgery. While a reduction in the risk of infections, length of ICU, and hospital stay was demonstrated in the dexamethasone group, no decrease in risk was observed with respect to stroke and postoperative mortality. In addition, dexamethasone was related to elevated postoperative glucose levels (*P* < 0.001) [55].

Due to the potential adverse effects associated with corticosteroids, the utilization of this therapy for prevention of POAF is not a standard therapy at this time.

#### 5.3.4. *ω*-3 Fatty Acids

*Ω*-3 Polyunsaturated fatty acids (*ω*-3-PUFAs) are parts of the biological membrane which produce a stabilizing effect. Additionally, *ω*-3-PUFAs have a positive effect on several ion channels and are known to reduce inflammation, oxidative stress, atrial electrical remodelling, and rarefy structural atria alternations.

A meta-analysis of 10 studies involving 1955 patients showed that *ω*-3-PUFAs was not effective in the prevention of POAF (OR 0.81; 95% CI 0.57 to 1.15; *P* = 0.24) [57]. This was also confirmed in a recent meta-analysis of 8 studies on 2717 patients (OR 0.85; 95% CI 0.72 to 1.00; *P* = 0.24) [58]. A recent multicenter RCT (OPERA-Study) consisting of 1516 patients who received either perioperative supplementation with *ω*-3-PUFAs or placebo also failed to demonstrate a risk reduction of POAF (OR 0.96; 95% CI 0.77 to 1.20; *P* = 0.74). *Ω*-3-PUFAs were not associated with major adverse events including stroke, postoperative, and cardiovascular mortality [56]. Current evidence suggests that short-term *ω*-3-PUFA use has no effect on POAF prevention after cardiac surgery.

## 6. Conclusion

Despite numerous management strategies, POAF still remains a major medical problem and poses serious risks and consequences.

The pathogenesis of POAF still remains not fully understood. The multiple wavelet re-entry theory in combination with certain biochemical mechanisms including neurohormonal activation, volume overload, and inflammation is likely the major contributing factor. Several risk factors have been described for the development of POAF. Recognizing these risk factors will aid physicians to take preventative strategy in high-risk patients.

Current guidelines suggest that when POAF persists for more than 48 hours, anticoagulation for 30 days with a vitamin K antagonist should be utilized to achieve an international normalizing ratio between 2.0 and 3.0. However, due to difficulty managing warfarin, new oral anticoagulants such as rivaroxaban, apixaban, and dabigatran are viewed as possible future options. Future studies to assess the risk-benefit ratio of these medications for the cardiac surgery population may increase the use of these medications in the near future.

Current evidence suggests that *β*-blocker therapy should be utilized during the perioperative setting in all patients when contraindications are absent. Amiodarone therapy could be supplemented in patients who have a high risk of developing POAF. Due to the known potential side effects, sotalol therapy should only be administrated to high risk patients and in the absence of contraindications. When high risk patients have a contraindication for these antiarrhythmic drugs, biatrial pacing could be considered. With regard to statins, it is reasonable to administrate prophylactic statin therapy in all patients who tolerate statins at least a week before cardiac surgery. Magnesium therapy, corticosteroids, and posterior pericardiotomy still remain controversial on whether the utilization of these interventions could achieve a risk reduction. Current evidence suggests that short-term *ω*-3-PUFA use has no effect on POAF prevention after cardiac surgery.

In prevention of POAF, understanding of the risk and pathophysiology and choosing adequate prophylactic option are the key.

## Figures and Tables

**Table 1:** Pre-, intra-, and postoperative (nonmodifiable and modifiable) clinical risk factors associated with POAF.

Preoperative risk factors	Intraoperative risk factors	Postoperative risk factors
*Nonmodifiable risk factors *	*Nonmodifiable risk factors *	*Nonmodifiable risk factors *
High age	Endotracheal tube insertion	Return to intensive care unit
Male gender	Intraoperative IABP	Ventilation longer than 24 hours
Previous cardiac surgery	Left ventricular venting	*Modifiable risk factors *
Valvular heart disease	Aortic cross-clamp time	Volume overload
Chronic lung disease	Extracorporeal circulation	Pneumonia
Chronic renal failure	Myocardial ischemia	Electrolyte imbalances
Left atrium enlargement	Venous cannulation	Imbalance of the autonomic nervous system
Left ventricular hypertrophy	*Modifiable risk factors *	Atrial extrasystole
*Modifiable risk factors *	Damage to the atrium	Increased postoperative adrenergic status
Withdrawal of *β*-blocker medication	Excess inotropic requirements	Increased afterload
History of AF	Acute volume change	Inflammation
Hypertension		Hypotension
Obesity		
Diabetes		
Metabolic syndrome		

IABP: Intra-Aortic Balloon Pump.

**Table 2:** Overview of the included nonpharmacological studies.

Intervention	Author	Ref.	Year	Design	Outcomes of interest	Study* N* Design	Patients *N *		Results	*P* value	NNT (95% CI)	Bottom line
							Intervention	Control	Odds ratio (95% CI)			
Posterior pericardiotomy	Arsenault et al.	[43]	2013	MA	POAF	6 RCTs	379	384	0.35 (0.18 to 0.67)	0.001	6 (4–8)	Posterior pericardiotomy is beneficial
					Lengte of hospital stay	3 RCTs	229	234	0.57 days (−1.99 to 3.12)	0.66	NA	No decrease in lengte of hospital stay
					Mortality	1 RCT	100	100	1.00 (0.06 to 16.44)	1.00	NA	No significant decrease in mortality
	Biancari and Mahar	[44]	2010	MA	POAF	6 RCTs	379	384	0.33 (0.16 to 0.69)	0.003	6 (4–8)	Posterior pericardiotomy is beneficial

Atrial pacing	Arsenault et al.	[43]	2013	MA	POAF	21 RCTs	1446	1487	0.47 (0.36 to 0.61)	<0.00001	8 (6–9)	Atrial pacing is beneficial
					Stroke	6 RCTs	419	413	0.72 (0.36 to 1.46)	0.36	NA	No significant decrease in stroke
					CV mortality	2 RCTs	98	100	0.0 (0.0 to 0.0)	<0.00001	NA	No significant decrease in CV mortality
					Length of stay	18 RCTs	742	783	−1.13 days (−1.72 to −0.55)	0.00015	NA	Shorter length of stay
	Burgess et al.	[45]	2006	MA	POAF	14 RCTs	923	962	0.60 (0.47–0.77)	<0.001	8 (6–9)	Atrial pacing is beneficial
					POAF in biatrial group	10 RCTs	367	387	0.44 (0.31–0.64)	<0.001	7 (5–10)	Biatrial pacing is beneficial
					Stroke	5 RCTs	268	282	0.61 (0.20 to 1.9)	0.48	NA	No significant decrease in stroke
					Length of stay	5 RCTs	268	282	−1.3 days (−2.55 to −0.08)	0.04	NA	Shorter length of stay

POAF: postoperative atrial fibrillation; NA: not applicable; NNT: number needed to treat; NNH: number needed to harm; hrs: hours; ref.: reference; postop: postoperative; preop: preoperative; RCT: randomized control trial; MA: meta-analysis.

**Table 3:** Overview of the included pharmacological studies.

Drug	Author	Ref.	Year	Design	Outcomes of interest	Study *N* Design	Patients *N *		Results	*P* value	NNT (95% CI)	Bottom line
							Intervention	Control	Odds Ratio (95% CI)			
*β*-Blockers	Arsenault et al.	[43]	2013	MA	POAF	33 RCTs	2294	2404	0.33 (0.26 to 0.43)	<0.00001	7 (6–8)	*β*- Blockers are beneficial
					Stroke	5 RCTs	774	780	1.34 (0.46 to 3.93)	0.59	81 (43−505)	No significant decrease in stroke
					Mortality	16 RCTs	1329	1342	0.87 (0.34 to 2.22)	0.70	NA	No significant decrease in mortality
					CV mortality	11 RCTs	1003	1008	0.98 (0.10 to 9.66)	0.99	NA	No significant decrease in CV mortality
					Length of stay	6 RCTs	844	832	−0.74 days (−1.48 to 0.00)	0.049	NA	Shorter length of stay
	Khan et al.	[46]	2013	MA	POAF	10 RCTs	1280	1276	0.50 (0.36 to 0.69)	<0.001	8 (6–11)	*β*-Blockers are beneficial

Amiodarone	Arsenault et al.	[43]	2013	MA	POAF	33 RCTs	2603	2799	0.43 (0.34 to 0.54)	<0.00001	8 (6–9)	Amiodarone is beneficial
					Stroke	14 RCTs	1523	1564	0.60 (0.35 to 1.02)	0.061	NA	No significant decrease in stroke
					Mortality	23 RCTs	2045	2132	1.08 (0.74 to 1.56)	0.70	NA	No significant decrease in mortality
					CV mortality	14 RCTs	1262	1253	0.93 (0.46 to 1.86)	0.83	NA	No significant decrease in CV mortality
					Length of stay	20 RCTs	1716	1781	−0.95 days (−1.37 to −0.52)	0.000013	NA	Significant decrease in length of stay
	Chatterjee et al.	[47]	2013	MA	POAF (oral-only)	8 RCTs	961	945	0.59 (0.49 to 0.70)	<0.00001	8 (6–11)	Amiodarone is beneficial
					POAF (iv.)	15 RCTs	1052	992	0.57 (0.48 to 0.75)	<0.00001	8 (6–10)	POAF independent with regard to the route,
					POAF preop administration	11 RCTs	1146	1067	0.55 (0.46 to 0.65)	<0.00001	7 (5–8)	timing of drug adm., and duration of treatment
					POAF postop administration	12 RCTs	867	850	0.50 (0.33 to 0.75)	0.0009	9 (6–14)	

	Arsenault et al.	[43]	2013	MA	POAF	11 RCTs	799	810	0.34 (0.26 to 0.43)	<0.00001	5 (4–6)	Sotalol is beneficial
					Stroke	1 RCT	63	65	0.34 (0.01 to 8.47)	0.51	NA	No significant decrease in stroke
					CV mortality	7 RCTs	475	489	0.0 (0.0 to 0.0)	<0.00001	NA	No significant decrease in CV mortality
					Length of stay	7 RCTs	455	456	−0.39 days (−0.77 to −0.02)	0.040	NA	Shorter length of stay
Sotalol	Kerin and Jacob	[48]	2011	MA	POAF (sotalol versus placebo)	5 RCTs	489	499	0.55 (0.45 to 0.67)	<0.001	6 (4–8)	Sotalol is beneficial
					POAF (sotalol versus no treatment)	6 RCTs	304	311	0.33 (0.24 to 0.46)	<0.001	4 (3–5)	Shorter length of stay
					POAF (sotalol versus *β*-blocker)	6 RCTs	488	555	0.60 (0.50 to 0.84)	<0.001	12 (8–28)	
					POAF preop administration	5 RCTs	389	400	0.55 (0.45 to 0.68)	<0.001	4 (3–5)	
					POAF postop administration	6 RCTs	404	410	0.39 (0.29 to 0.51)	<0.001	5 (3–5)	
					Length of stay	5 RCTs	339	349	−0.5 days (−1.06 to −0.05)	<0.072	NA	

POAF: postoperative atrial fibrillation; NA: not applicable; NNT: number needed to treat; NNH: number needed to harm; ref.: reference; postop: postoperative; preop: preoperative; iv.: intravenous; RCT: randomized control trial; MA: meta-analysis.

**Table 4:** Overview of the included upstream therapy studies.

Drug	Author	Ref.	Year	Design	Outcomes of Interest	Study* N * Design	Patients *N *		Results	*P* value	NNT (95% CI)	Bottom line
							Intervention	Control	Odds Ratio (95% CI)			
Magnesium	Arsenault et al.	[43]	2013	MA	POAF	21 RCTs	1567	1421	0.55 (0.41 to 0.73)	<0.0001	11 (8–15)	Magnesium is beneficial
					Stroke	3 RCTs	380	380	0.33 (0.03 to 3.20)	0.34	NA	No significant decrease in stroke
					Mortality	12 RCTs	907	857	0.83 (0.31 to 2.24)	0.72	NA	No significant decrease in mortality
					CV mortality	9 RCTs	502	460	0.53 (0.09 to 3.13)	0.49	NA	No significant decrease in CV mortality
					Length of stay	9 RCTs	798	791	0.05 days (−0.47 to 0.57)	0.86	NA	No significant decrease in length of stay
	Shepherd et al.	[49]	2008	MA	POAF	15 RCTs	1070	1031	0.65 (0.53 to 0.79)	<0.0001	13 (8–22)	Magnesium is beneficial

Statins	Liakopoulos et al.	[50]	2012	MA	POAF	11 RCTs	422	419	0.40 (0.29 to 0.55)	<0.00001	7 (4–9)	Statins are beneficial
					Mortality	1 RCT	101	99	0.98 (0.14 to 7.10)	0.98	NA	No significant decrease in mortality
					Stroke	2 RCTs	133	131	0.70 (0.14 to 3.63)	0.67	NA	No significant decrease in stroke
					ICU stay	7 RCTs	263	258	−3.39 hrs (−5.77 to −1.01)	0.0052	NA	Shorter ICU and length of stay
					Length of stay	8 RCTs	442	435	−0.48 days (−0.85 to −0.11)	0.011	NA	
	Chopra et al.	[51]	2012	MA	POAF	9 RCTs	467	466	0.56 (0.45 to 0.69)	<0.0001	7 (5–9)	Statins are beneficial
	Chen et al.	[52]	2010	MA	POAF	8 RCTs	326	325	0.57 (0.45–0.72)	0.0006	6 (4–10)	Statins are beneficial
					ICU stay	5 RCTs	167	164	−0.17 hrs (−0.37 to 0.03)	NA	NA	Shorter ICU and length of stay
					Length of stay	6 RCTs	687		−0.66 days (−1.01 to −0.30)	NA	NA

Corticosteroids	Ho and Tan	[53]	2009	MA	POAF	17 RCTs	752	757	0.74 (0.63 to 0.86)	0.0001	10 (7–19)	Corticosteroids are beneficial
					Infection	22 RCTs	806	802	0.93 (0.61 to 1.41)	0.73	NA	No increase in infection (but more hyperglycemia needing insulin, 28% RR)
					Mortality	35 RCTs	1407	1379	0.72 (0.45 to 1.14)	0.16	NA	Increased hyperglycemia when utilization of high doses
					Hyperglycemia	9 RCTs	255	248	1.49 (1.11 to 2.01)	0.009	NNH = 9 (5–25)	Dose did not affect the outcome
	Dieleman et al.	[54]	2011	MA	POAF	17 RCTs	694	695	0.60 (0.46 to 0.78)	0.00016	13 (8–28)	Corticosteroids are beneficial
					Stroke	10 RCTs	538	514	0.70 (0.33 to 1.48)	0.35	NA	No significant impact on stroke, mortality,
					Mortality	17 RCTs	1036	976	1.12 (0.65 to 1.92)	0.68	NA	and infections
					Infections	15 RCTs	744	743	0.86 (0.56 to 1.31)	0.47	NA	Shorter ICU and hospital stay
					ICU stay	25 RCTs	605	610	−2.32 hrs (−2.84 to −1.81)	<0.00001	NA	
					Hospital stay	15 RCTs	312	313	−0.40 days (−0.65 to −0.15)	0.0017	NA	
	Dieleman et al.	[55]	2012	RCT	POAF	1 RCT	2235	2247	0.94 (0.87 to 1.02)	0.14	NA	Corticosteroids are not beneficial
					Stroke	1 RCT	2235	2247	0.91 (0.55 to 1.50)	0.72	NA	Decreased risk of infection
					Mortality	1 RCT	2235	2247	0.92 (0.57 to 1.49)	0.73	NA	No significant impact on stroke
					Infection	1 RCT	2235	2247	0.64 (0.54 to 0.75)	<0.001	19 (14–29)	No significant impact on mortality
					Length of stay	1 RCT	2235	2247	NA	0.009	NA	Shorter ICU and hospital stay
					ICU stay	1 RCT	2235	2247	NA	<0.001	NA	Increased risk of hyperglycemia
					Hyperglycemia	1 RCT	2235	2247	NA	<0.001	NA	

*ω*-3-PUFAs	Mozaffarian et al.	[56]	2012	RCT	POAF	1 RCT	758	758	0.96 (0.77 to 1.20)	0.74	NA	*ω*-3-PUFAs are not beneficial
					Stroke	1 RCT	758	758	0.45 (0.13 to 1.51)	0.18	NA	No significant decrease in stroke
					Mortality	1 RCT	758	758	0.53 (0.23 to 1.26)	0.14	NA	No significant decrease in (CV) mortality
					CV mortality	1 RCT	758	758	NA	0.08	NA	No significant decrease in hospital stay
					Hospital stay	1 RCT	758	758	NA	0.48	NA	
	Liu et al.	[57]	2011	MA	POAF	10 RCTs	977	978	0.81 (0.57 to 1.15)	0.24	NA	*ω*-3-PUFAs are not beneficial
	Mozaffarian et al.	[58]	2013	MA	POAF	8 RCTs	2717	NA	0.85 (0.72 to 1.00)	0.24	NA	*ω*-3-PUFAs are not beneficial

POAF: postoperative atrial fibrillation; NA: Not applicable; NNT: number needed to treat; NNH: number needed to harm; hrs: hours; ref.: reference; postop: postoperative; preop: preoperative; RCT: randomized control trial; MA: meta-analysis.

## References

[1] Shrivastava R, Smith B, Caskey D, Reddy P (2009). Atrial fibrillation after cardiac surgery: does prophylactic therapy decrease adverse outcomes associated with atrial fibrillation. *Journal of Intensive Care Medicine*.

[2] Hakala T, Halonen J, Mäkinen K, Hartikainen J (2013). Prevention of atrial fibrillation after cardiac surgery. *Scandinavian Journal of Surgery*.

[3] Mathew JP, Fontes ML, Tudor IC (2004). A multicenter risk index for atrial fibrillation after cardiac surgery. *The Journal of the American Medical Association*.

[4] Gillespie EL, White CM, Kluger J, Sahni J, Gallagher R, Coleman CI (2005). A hospital perspective on the cost-effectiveness of *β*-blockade for prophylaxis of atrial fibrillation after cardiothoracic surgery. *Clinical Therapeutics*.

[5] Gillespie EL, White CM, Kluger J, Rancourt JA, Gallagher R, Coleman CI (2006). Cost-effectiveness of amiodarone for prophylaxis of atrial fibrillation after cardiothoracic surgery. *Pharmacotherapy*.

[6] Filardo G, Hamilton C, Hebeler RF, Hamman B, Grayburn P (2009). New-onset postoperative atrial fibrillation after isolated coronary artery bypass graft surgery and long-term survival. *Circulation: Cardiovascular Quality and Outcomes*.

[7] Bramer S, van Straten AHM, Soliman Hamad MA, Berreklouw E, Martens EJ, Maessen JG (2010). The impact of preoperative atrial fibrillation on early and late mortality after coronary artery bypass grafting. *European Journal of Cardio-Thoracic Surgery*.

[8] Kalavrouziotis D, Buth KJ, Vyas T, Ali IS (2009). Preoperative atrial fibrillation decreases event-free survival following cardiac surgery. *European Journal of Cardio-Thoracic Surgery*.

[9] Schulenberg R, Antonitsis P, Stroebel A, Westaby S (2010). Chronic atrial fibrillation is associated with reduced survival after aortic and double valve replacement. *Annals of Thoracic Surgery*.

[10] El-Chami MF, Kilgo P, Thourani V (2010). New-onset atrial fibrillation predicts long-term mortality after coronary artery bypass graft. *Journal of the American College of Cardiology*.

[11] Cox JL (1993). A perspective of postoperative atrial fibrillation in cardiac operations. *Annals of Thoracic Surgery*.

[12] Aldhoon B, Melenovský V, Peichl P, Kautzner J (2010). New insights into mechanisms of atrial fibrillation. *Physiological Research*.

[13] Nattel S, Burstein B, Dobrev D (2008). Atrial remodeling and atrial fibrillation: mechanisms and implications. *Circulation. Arrhythmia and Electrophysiology*.

[14] Jalife J (2011). Déjà vu in the theories of atrial fibrillation dynamics. *Cardiovascular Research*.

[15] Haïssaguerre M, Jaïs P, Shah DC (1998). Spontaneous initiation of atrial fibrillation by ectopic beats originating in the pulmonary veins. *The New England Journal of Medicine*.

[16] Workman AJ (2010). Cardiac adrenergic control and atrial fibrillation. *Naunyn-Schmiedeberg’s Archives of Pharmacology*.

[17] Ravelli F, Allessie M (1997). Effects of atrial dilatation on refractory period and vulnerability to atrial fibrillation in the isolated Langendorff-perfused rabbit heart. *Circulation*.

[18] Calkins H, El-Atassi R, Leon A (1992). Effect of the atrioventricular relationship on atrial refractoriness in humans. *Pacing and Clinical Electrophysiology*.

[19] Nardi F, Diena M, Caimmi PP (2012). Relationship between left atrial volume and atrial fibrillation following coronary artery bypass grafting. *Journal of Cardiac Surgery*.

[20] Kalus JS, Caron MF, White CM (2004). Impact of fluid balance on incidence of atrial fibrillation after cardiothoracic surgery. *American Journal of Cardiology*.

[21] Kaireviciute D, Blann AD, Balakrishnan B (2010). Characterisation and validity of inflammatory biomarkers in the prediction of post-operative atrial fibrillation in coronary artery disease patients. *Thrombosis and Haemostasis*.

[22] Tselentakis EV, Woodford E, Chandy J, Gaudette GR, Saltman AE (2006). Inflammation effects on the electrical properties of atrial tissue and inducibility of postoperative atrial fibrillation. *Journal of Surgical Research*.

[23] Aranki SF, Shaw DP, Adams DH (1996). Predictors of atrial fibrillation after coronary artery surgery: current trends and impact on hospital resources. *Circulation*.

[24] Afilalo J, Rasti M, Ohayon SM, Shimony A, Eisenberg MJ (2012). Off-pump vs. on-pump coronary artery bypass surgery: an updated meta-analysis and meta-regression of randomized trials. *European Heart Journal*.

[25] Creswell LL, Alexander JC, Ferguson TB, Lisbon A, Fleisher LA (2005). Intraoperative interventions: American College of Chest Physicians guidelines for the prevention and management of postoperative atrial fibrillation after cardiac surgery. *Chest*.

[26] Angelini GD, Penny WJ, El-Ghamary F (1987). The incidence and significance of early pericardial effusion after open heart surgery. *European Journal of Cardio-Thoracic Surgery*.

[27] Bryan AJ, Angelini GD (1990). Pericardial effusion after open heart surgery. *Thorax*.

[28] Thorén E, Hellgren L, Jidéus L, Ståhle E (2012). Prediction of postoperative atrial fibrillation in a large coronary artery bypass grafting cohort. *Interactive CardioVascular and Thoracic Surgery*.

[29] Zaman AG, Archbold RA, Helft G, Paul EA, Curzen NP, Mills PG (2000). Atrial fibrillation after coronary artery bypass surgery: a model for preoperative risk stratification. *Circulation*.

[30] Villareal RP, Hariharan R, Liu BC (2004). Postoperative atrial fibrillation and mortality after coronary artery bypass surgery. *Journal of the American College of Cardiology*.

[31] Sedrakyan A, Wu AW, Parashar A, Bass EB, Treasure T (2006). Off-pump surgery is associated with reduced occurrence of stroke and other morbidity as compared with traditional coronary artery bypass grafting: a meta-analysis of systematically reviewed trials. *Stroke*.

[32] Cheng DC, Bainbridge D, Martin JE, Novick RJ (2005). Does off-pump coronary artery bypass reduce mortality, morbidity, and resource utilization when compared with conventional coronary artery bypass? A meta-analysis of randomized trials. *Anesthesiology*.

[33] Lamy A, Devereaux PJ, Prabhakaran D (2012). Off-pump or on-pump coronary-artery bypass grafting at 30 days. *The New England Journal of Medicine*.

[34] Fuster V, Rydén LE, Cannom DS (2011). 2011 ACCF/AHA/HRS focused updates incorporated into the ACC/AHA/ESC 2006 guidelines for the management of patients with atrial fibrillation: a report of the American College of Cardiology Foundation/American Heart Association Task Force on practice guidelines. *Circulation*.

[35] Sprigg N, Selby J, Fox L, Berge E, Whynes D, Bath PM (2013). Very low quality of life after acute stroke: data from the efficacy of nitric oxide in stroke trial. *Stroke*.

[36] Saxena A, Dinh DT, Smith JA, Shardey GC, Reid CM, Newcomb AE (2012). Usefulness of postoperative atrial fibrillation as an independent predictor for worse early and late outcomes after isolated coronary artery bypass grafting (multicenter Australian study of 19,497 patients). *American Journal of Cardiology*.

[37] Wyse DG, Waldo AL, DiMarco JP (2002). A comparison of rate control and rhythm control in patients with atrial fibrillation. *The New England Journal of Medicine*.

[38] Epstein AE, Alexander JC, Gutterman DD, Maisel W, Wharton JM (2005). Anticoagulation: American College of Chest Physicians guidelines for the prevention and management of postoperative atrial fibrillation after cardiac surgery. *Chest*.

[39] Connolly SJ, Ezekowitz MD, Yusuf S (2009). Dabigatran versus warfarin in patients with atrial fibrillation. *The New England Journal of Medicine*.

[40] Wann LS, Curtis AB, Ellenbogen KA (2011). 2011 ACCF/AHA/HRS focused update on the management of patients with atrial fibrillation (update on Dabigatran): a report of the American College of Cardiology Foundation/American Heart Association Task Force on practice guidelines. *Circulation*.

[41] Patel MR, Mahaffey KW, Garg J (2011). Rivaroxaban versus warfarin in nonvalvular atrial fibrillation. *The New England Journal of Medicine*.

[42] Granger CB, Alexander JH, McMurray JJ (2011). Apixaban versus warfarin in patients with atrial fibrillation. *The New England Journal of Medicine*.

[43] Arsenault KA, Yusuf AM, Crystal E (2013). Interventions for preventing post-operative atrial fibrillation in patients undergoing heart surgery. *The Cochrane Database of Systematic Reviews*.

[44] Biancari F, Mahar MAA (2010). Meta-analysis of randomized trials on the efficacy of posterior pericardiotomy in preventing atrial fibrillation after coronary artery bypass surgery. *The Journal of Thoracic and Cardiovascular Surgery*.

[45] Burgess DC, Kilborn MJ, Keech AC (2006). Interventions for prevention of post-operative atrial fibrillation and its complications after cardiac surgery: a meta-analysis. *European Heart Journal*.

[46] Khan MF, Wendel CS, Movahed MR (2013). Prevention of post-coronary artery bypass grafting (CABG) atrial fibrillation: efficacy of prophylactic beta-blockers in the modern era: a meta-analysis of latest randomized controlled trials. *Annals of Noninvasive Electrocardiology*.

[47] Chatterjee S, Sardar P, Mukherjee D, Lichstein E, Aikat S (2013). Timing and route of amiodarone for prevention of postoperative atrial fibrillation after cardiac surgery: a network regression meta-analysi. *Pacing and Clinical Electrophysiology*.

[48] Kerin NZ, Jacob S (2011). The efficacy of sotalol in preventing postoperative atrial fibrillation: a meta-analysis. *American Journal of Medicine*.

[49] Shepherd J, Jones J, Frampton GK, Tanajewski L, Turner D, Price A (2008). Intravenous magnesium sulphate and sotalol for prevention of atrial fibrillation after coronary artery bypass surgery: a systematic review and economic evaluation. *Health Technology Assessment*.

[50] Liakopoulos OJ, Kuhn EW, Slottosch I, Wassmer G, Wahlers T (2012). Preoperative statin therapy for patients undergoing cardiac surgery. *The Cochrane Database of Systematic Reviews*.

[51] Chopra V, Wesorick DH, Sussman JB (2012). Effect of perioperative statins on death, myocardial infarction, atrial fibrillation, and length of stay: a systematic review and meta-analysis. *Archives of Surgery*.

[52] Chen WT, Krishnan GM, Sood N, Kluger J, Coleman CI (2010). Effect of statins on atrial fibrillation after cardiac surgery: a duration- and dose-response meta-analysis. *The Journal of Thoracic and Cardiovascular Surgery*.

[53] Ho KM, Tan JA (2009). Benefits and risks of corticosteroid prophylaxis in adult cardiac surgery a dose-response meta-analysis. *Circulation*.

[54] Dieleman JM, van Paassen J, van Dijk D (2011). Prophylactic corticosteroids for cardiopulmonary bypass in adults. *The Cochrane Database of Systematic Reviews*.

[55] Dieleman JM, Nierich AP, Rosseel PM (2012). Intraoperative high-dose dexamethasone for cardiac surgery: a randomized controlled trial. *The Journal of the American Medical Association*.

[56] Mozaffarian D, Marchioli R, Macchia A (2012). Fish oil and postoperative atrial fibrillation: the Omega-3 Fatty Acids for Prevention of Post-operative Atrial Fibrillation (OPERA) randomized trial. *The Journal of the American Medical Association*.

[57] Liu T, Korantzopoulos P, Shehata M, Li G, Wang X, Kaul S (2011). Prevention of atrial fibrillation with omega-3 fatty acids: a meta-analysis of randomised clinical trials. *Heart*.

[58] Mozaffarian D, Wu JH, de Oliveira Otto MC (2013). Fish oil and post-operative atrial fibrillation: a meta-analysis of randomized controlled trials. *Journal of the American College of Cardiology*.

[59] Yorgancioğlu C, Farsak B, Tokmakoğlu H, Günaydin S (2000). An unusual experience with posterior pericardiotomy. *European Journal of Cardio-Thoracic Surgery*.

[60] Patel AA, White CM, Gillespie EL, Kluger J, Coleman CI (2006). Safety of amiodarone in the prevention of postoperative atrial fibrillation: a meta-analysis. *American Journal of Health-System Pharmacy*.

[61] Arstall MA, Hii JTY, Lehman RG, Horowitz JD (1992). Sotalol-induced torsade de pointes: management with magnesium infusion. *Postgraduate Medical Journal*.

[62] Antonaccio MJ, Lessem JN, Soyka LF (1984). Sotalol, hypokalaemia, syncope, and torsade de pointes. *British Heart Journal*.

[63] Hillis LD, Smith PK, Anderson JL (2011). 2011 ACCF/AHA guideline for coronary artery bypass graft surgery: a report of the American College of Cardiology Foundation/American Heart Association Task Force on practice guidelines. *Circulation*.

[64] Aglio LS, Stanford GG, Maddi R, Boyd JL, Nussbaum S, Chernow B (1991). Hypomagnesemia is common following cardiac surgery. *Journal of Cardiothoracic and Vascular Anesthesia*.

[65] Booth JV, Phillips-Bute B, McCants CB (2003). Low serum magnesium level predicts major adverse cardiac events after coronary artery bypass graft surgery. *American Heart Journal*.

[66] Klevay LM, Milne DB (2002). Low dietary magnesium increases supraventricular ectopy. *American Journal of Clinical Nutrition*.

